# Supplementary material for: Sorting at embryonic boundaries requires high heterotypic interfacial tension
Source: Nat Commun. 2017 Jul 31;8:157. doi: 10.1038/s41467-017-00146-x (PMC5537356; doi:10.1038/s41467-017-00146-x)
Supplement: Supplementary file 2 — Supplementary Software 1 [file 41467_2017_146_MOESM2_ESM.zip › PottsModel/SrcPottsModel/doc/index-files/index-6.html]

F-Index


JavaScript is disabled on your browser.


Skip navigation links


- Overview
- Package
- Class
- Use
- Tree
- Deprecated
- Index
- Help

- Prev Letter
- Next Letter

- Frames
- No Frames

- All Classes

A B C D E F G H I J K L M N O P R S T U V W 


## F

FiniteVector - Class in model


FiniteVector() - Constructor for class model.FiniteVector


FiniteVector(FiniteVector) - Constructor for class model.FiniteVector


FiniteVector(Point, double, double) - Constructor for class model.FiniteVector


FiniteVector(Point, Point) - Constructor for class model.FiniteVector


first - Variable in class model.Pair


FourByFourSimulation - Class in simulations


FourByFourSimulation() - Constructor for class simulations.FourByFourSimulation


fromRGB(Color) - Static method in class utils.HSLColor
:   Convert a RGB Color to it corresponding HSL values.

A B C D E F G H I J K L M N O P R S T U V W

Skip navigation links


- Overview
- Package
- Class
- Use
- Tree
- Deprecated
- Index
- Help

- Prev Letter
- Next Letter

- Frames
- No Frames

- All Classes
